# Supplementary material for: StressMatic: A Novel Automated System to Induce Depressive- and Anxiety-like Phenotype in Rats
Source: Cells. 2023 Jan 20;12(3):381. doi: 10.3390/cells12030381 (PMC9913774; doi:10.3390/cells12030381)
Supplement: Supplementary file 1 [file cells-12-00381-s001.zip › cells-2145320-supplementary.pdf]

Supplementary methods

1. Rack development and optimization

1.1 General features and Mechanical details

This chronic stress equipment is programmed through a computer custom software controlled by a touch-screen (**Figure 1, yellow box**).

The uCMS is a labor-intensive protocol, particularly the manipulation of the cages during some stressors. To overcome this issue, a metallic structure hangs the cage lids, allowing the opening of 3 cage lids at once. This feature (**Figure 1, cage row 2**) is a user-friendly structure very useful to stressors that involve manipulation of the cages by the experimenter, particularly to perform stressors of deprivation/inaccessibility of food or water as well as to perform our proposed confinement stressor. Also, it is helpful for stressors dependent on animals' manipulation, like overcrowding or switch-cage.

1.2 Stressors categories

The stressors were divided into 3 categories: fully automated, partially automated, and manual, as mentioned in the Methods section (**Table 1**). In this section, we provide additional details regarding the implementation of these stressors.

1.2.1 Fully Automated stressors

The rack performs fully automated stressors without the experimenter's intervention. This category encompasses motor-dependent stressors (A), water/air supply-dependent stressors (B), light-dependent stressors (C, D, E), and the sound-dependent stressor (F).

Tilted-cage (A)

In the original protocol, the cages are placed on a table on top of the cage grid, to enable their inclination for long periods. Our automated equipment has a motor incorporated to lift and hold the cage base at a specific angle (**Figure 1, cage row 3**). The tilt angle is programmed at the beginning of the protocol.

Damp bedding (B)

In the manual protocol, water is added to the cages individually. Our automated system enables the provision of a uniform and simultaneous water supply to each cage through a tube system linked to the cage lids. The quantity of water released can be programmed (**Figure 1, blue tubes**). For water distribution, we selected tubes with a diameter of 16mm to achieve an equal distribution of water and be capable of supplying water until the highest and distant corner of the rack. Regarding the choice of material, a low-density polyethylene tube was selected. This material is not toxic or harmful to the animals and does not erode with water.

Additionally, the system also has the particularity to supply air (using the same piping used for water) to purge the system after water-involving stressors. The objective is to dry the piping after each damp bedding to prevent the growth of fungi and bacteria.

Regarding the architecture of the structure, water is provided only in two sets of rows (1st, 2nd line, and 3rd, 4th line) to minimize the water volume error for each cage. Finally, to equalize the volume of water per cage, mini taps were introduced near the tube exit to regulate the flow.

It must be highlighted that at the beginning of each protocol, the system needs to be refined and pre-tested and mini-taps must be adjusted to confirm the standardization of volumes. Also,

to have equal water volumes in each cage, the pipes need to be filled with water. Before each protocol, a priming process must be performed to completely fill the tubes for some seconds.

Light-dependent stressors

Several stressors (C, D, E) are light-dependent. The system includes and controls a complete room light setting (Figure 1, gray wall). Lights and stressor settings are programmed in parallel flows in the software, as they are used simultaneously with other stressors, for example, an inverted light cycle with a tilted cage. For the E stressor, strobe lights were incorporated into the computer box (**Figure 1, yellow box**).

Sound-dependent stressor

Lastly, to perform the startle-noise stressor (F) two speakers were added to the system for software-controlled delivery of startle sounds.

1.2.2 Partially automated stressors

Confinement to a restricted space (K)

Partially automated stressors are not performed by the system but are eased compared to the manual protocol to reduce the intervention and workload of the experimenter. In the manual protocol, confinement is achieved by putting 3 animals in a plastic box; in this alternative version of the protocol, an acrylic T-shaped object was designed to be inserted in the home cage, reducing the available space and confining the animals.

In developing this stressor, measuring the volume of the plastic boxes used in the manual protocol (3L) was necessary. Our device was designed to produce the same effect as the manual stressor.

1.2.3 Manual stressors

Food deprivation followed by exposure to inaccessible food (G)

In the original protocol, at the end of the day, the food is removed from the cages by the experimenter. In the morning, a container is inserted that allows the animals to smell but not eat the food (for 1 hour). To reduce workload, a custom food dispenser was designed and produced. This food restrictor allows the animal to access or not access the food just by a 180-degree rotation.

All other manual stressors were performed manually, including H, I, J.

Table S1. Normality tests for all statistical analyses.

Figure ID	Passed normality test?	Statistics		
		CT	uCMS	auCMS
2B	Yes	K2=0.03921, p-value=0.9806	K2=5.299, p-value=0.0707	K2=5.312, p-value=0.0702
2C	Yes	W=0.9488, p-value=0.7185	W=0.9731, p-value=0.9201	W=0.9565, p-value=0.7884
3B	Yes	K2=1.878, p-value=0.3910	K2=0.8005, p-value=0.6702	K2=1.062, p-value=0.5881
3C1	No	KS=1.000, p-value<0.0001	KS=0.2591, p-value>0.1000	KS=0.2445, p-value>0.1000
3C2	No	W=0.8771, p-value=0.1766	W=0.6647, p-value=0.0009	W=0.9153, p-value=0.3930
3C3	Yes	LC: W=0.9572, p-value=0.7833 RC: W=0.9115, p-value=0.3645	-	-
3C3	Yes	-	LC: W=0.8857, p-value=0.2135 RC: W=0.9420, p-value=0.6310	-
3C3	Yes	-	-	LC: W=0.9422, p-value=0.6330RC: W=0.8634, p-value=0.1298
3D1	Yes	K2=2.781, p-value=0.2489	K2=5.568, p-value=0.0618	K2=2.637, p-value=0.2675
3E1	No	W=0.8100, p-value=0.0366	W=0.9263, p-value=0.4827	W=0.8155, p-value=0.4180
3E2	No	W=0.8253, p-value=0.0531	W=0.7860, p-value=0.0202	W=0.9360, p-value=0.5723
3F1	Yes	W=0.9075, p-value= 0.3368	W=0.8867, p-value=0.2178	W=0.9339, p-value=0.5845
3G1	Yes	W=0.9271, p-value=0.4899	W=0.8631, p-value=0.1289	W=0.9525, p-value=0.7355
3G2	Yes	W=0.8926, p-value=0.2472	W=0.9520, p-value=0.7309	W=0.9460, p-value=0.6708
4A1	No	Nadir: W=0.7219, p-value=0.0064; Zenith: W=0.9415, p-value=0.6261	-	-

4A1	Yes	-	Nadir: W=0.9398, p-value=0.5793;<br>Zenith: W=0.9569, p-value=0.7658	-
4A1	Yes	-	-	Nadir: W=0.9272, p-value=0.3514;<br>Zenith: W=0.8979, p-value=0.1742
4A2	Yes	Nadir: W=0.9399, p-value=0.2890;<br>Zenith: W=0.9279, p-value=0.1784	-	-
4A2	Yes	-	Nadir: KS=0.1582, p-value=0.0614;<br>Zenith: KS=0.1498; p-value>0.1000	-
4A2	Yes	-	-	Nadir: KS=0.1656, p-value>0.1000;<br>Zenith: KS=0.1427; p-value>0.1000
4B	Yes	K2=1.095, p-value=0.5785	K2=3.090, p-value=0.2134	K2=1.347, p-value=0.5098
